# High tissue expression of TLRs combined with high density of tumor infiltrating lymphocytes predicts a better prognosis in colorectal cancer patients

**DOI:** 10.1371/journal.pone.0280085

**Published:** 2023-01-17

**Authors:** Ines Beilmann-Lehtonen, Jussi Kasurinen, Jaana Hagström, Tuomas Kaprio, Camilla Böckelman, Caj Haglund

**Affiliations:** 1 Department of Surgery, University of Helsinki and Helsinki University Hospital, Helsinki, Finland; 2 Translational Cancer Medicine Research Program, Faculty of Medicine, University of Helsinki, Helsinki, Finland; 3 Department of Pathology, University of Helsinki and Helsinki University Hospital, Helsinki, Finland; 4 Department of Oral Pathology and Radiology, University of Turku, Turku, Finland; Texas Tech University Health Science, Lubbock, UNITED STATES

## Abstract

**Background:**

Colorectal cancer causes 935,000 cancer deaths yearly. High local immune cell infiltration serves as a positive prognostic factor in CRC. Toll-like receptors (TLRs) induce innate immune responses and lead to adaptive immune system activation. TLRs play protumorigenic and antitumorigenic roles. We aimed to explore the relationship between TLR immunoexpressions and the infiltration densities of T-lymphocytes in CRC.

**Methods:**

Immunohistochemical TLR2, TLR4, TLR5, and TLR7 positivity and the density of CD3- and CD8-positive cells in tumoral and stromal tissue were evaluated from the tissue microarray slides of 549 consecutive CRC surgical patients treated at Helsinki University Hospital, Finland, between 1998 and 2005. We calculated the associations and correlations using Pearson’s chi-square and Spearman’s correlation tests, generating survival curves using the Kaplan–Meier method.

**Results:**

Positive intratumoral CD3 and CD8 densities associated with a high TLR2 expression (*p* < 0.001 and *p* = 0.001, respectively) and a high TLR4 expression (*p* = 0.013 and *p* = 0.025). A low TLR5 immunoexpression associated with negative intratumoral CD3 (*p* = 0.001) and CD8 (*p* = 0.011) and a low stromal CD3 (*p* = 0.001). No association or correlation emerged between TLR7 immunoexpression and CD3 or CD8 cell density. A low CD3–CD8 tumor–stroma index indicated a worse prognosis among all TLR subgroups, except the TLR7-negative subgroup.

**Conclusions:**

We detected significant associations and correlations between high tissue TLR2, TLR4, and TLR5 immunoexpressions and high densities of CD3- and CD8-positive cells. Combining these markers may improve the prognostic evaluation of CRC patients.

## Introduction

Colorectal cancer (CRC) caused 935,000 cancer deaths in 2020, making it the second leading cause of cancer deaths worldwide [1], with global incidence expected to rise rapidly from 1.9 million in 2018 to 2.2 million new cases in 2030 [1, 2]. While treatments have improved, currently, 17% of stage II and 36% of stage III CRC patients experience recurrence within five years [3].

Chronic local inflammation contributes to the development of CRC [4]. CRC patients with a high C-reactive protein (CRP) level [5, 6], a high modified Glasgow prognostic score (GPS) [7, 8], or a high neutrophil–lymphocyte ratio (NLR) [9], all markers of an elevated systemic inflammatory response (SIR), exhibit a worse prognosis. Yet, a high local immune cell infiltration in different areas of the tumor leads to a better outcome by activating antitumorigenic immune processes [10].

Toll-like receptors (TLRs) are transmembranous proteins that recognize several structures of microbial origin, rendering them crucial in initiating innate immune responses against pathogens [11–13]. In addition to recognizing pathogen-associated molecular patterns (PAMPs), TLRs recognize host-originating damage-associated molecular patterns (DAMPs), released during tissue damage [13]. TLRs are expressed by intestinal and respiratory epithelial cells, innate immune cells such as natural killer cells (NKs), macrophages, dendritic cells, and monocytes, but also by adaptive immune cells such as B-cells and regulatory T-cells [13, 14]. Furthermore, tumor cells can express different TLRs [11, 14]. TLRs are crucial to initiating adaptive immune responses, since dendritic cells need activating signals from TLRs after PAMP recognition to mature and activate naive CD4-positive and CD8-positive T-cells [12, 15].

In malignancies, TLRs may promote both procancerous and antitumorigenic factors [14, 16]. We previously demonstrated that CRC patients with a high tissue TLR2 [17, 18] a high tissue TLR5 [18, 19], and a high tissue TLR7 immunoexpression [19] experience a better prognosis. Dukes B CRC patients with a high TLR4 immunoexpression, however, exhibit a worse prognosis [17].

Solid tumors are infiltrated with a multitude of immune cells, consisting among others of different types of T-lymphocytes. Particularly high densities of tumor-infiltrating lymphocytes (TILs) expressing CD3 or CD8 T-cell receptors on their surfaces indicate a good prognosis in several malignancies, including CRC [20, 21]. CD3, a T-cell co-receptor expressed in all developmental stages of T-cells, is needed for the activation of CD8- and CD4-positive naive cells [22]. Furthermore, CD8 is predominantly expressed only in cytotoxic T-cells, crucial in adaptive immune responses against pathogens and tumors since they recognize and eliminate cells that introduce foreign antigens [22, 23]. TLRs take part in T-cell development and differentiation [15].

The intratumoral and stromal densities of CD3- and CD8-positive immune cells can be used to determine the *immunoscore* and other indices that mirror the adaptive antitumoral immune response. Specifically, a high *immunoscore* indicated a better survival in various studies [24, 25]. While immunosubtyping patients according to only CD3 or CD8 densities is simpler, further studies are needed to determine whether both lymphocyte subgroups offer any additional prognostic value [26, 27]. In addition to the TNM stage [28], immunosubtyping of CRC patients might help to select patients for more targeted treatment [29, 30].

Here, we aimed to evaluate the possible relationship between the expressions of the local innate response markers TLR2, TLR4, TLR5, and TLR7 and the local adaptive immune response markers stromal and tumoral CD3 and CD8 densities in CRC patients.

## Materials and methods

### Patients

This retrospective study consisted of 549 consecutive patients treated surgically for CRC in the Department of Surgery, Helsinki University Hospital, Finland, between 1998 and 2005. The median age was 69.2 [interquartile range (IQR) 59.2–77.4], and 52.6% of patients were male. The median follow-up time was 6.44 years (IQR 2.00–14.85) and 379 patients (69%) died by the end of follow-up, 192 of whom (35%) died due to CRC. For cancer staging, we used the TNM sixth edition [31] whereby 108 patients (19.7%) had stage I, 153 (27.9%) stage II, 201 (36.7%) stage III, and 86 (15.7%) stage IV disease. The clinicopathological characteristics are demonstrated in S1 Table.

Clinical data were retrieved from medical records. Survival data and cause of death information were provided by the Population Register Center of Finland and Statistics Finland. The Surgical Ethics Committee of the Helsinki University Hospital approved the study protocol (Dnro HUS 226/E6/06, extension TMK02 §66 17.4.2013) and permission to study the archived tissue samples without requiring individual informed consent from each patient was granted by the National Supervisory Authority of Health and Welfare (Valvira Dnro 10041/06.01.03.01/2012).

### Preparation of tissue samples

Representative areas of the formalin-fixed and paraffin-embedded surgical tumor samples, provided by the Department of Pathology at the University of Helsinki, were prepared by an experienced pathologist (JH) on hematoxylin- and eosin-stained slides. Using a semiautomatic tissue arrayer (Beecher Instruments Inc., Silver Spring, MD, USA), 1.0-mm cores were taken from each tumor block and embedded in tissue microarray (TMA) paraffin blocks, cut into 4-μm sections as described previously [32].

### Immunohistochemistry for TLRs

The immunohistochemistry for TLRs is described in detail elsewhere [19]. For each TLR, we used the same staining protocol, using the following primary antibodies: TLR3 rabbit polyclonal (sc-10740, Santa Cruz Biotechnology, Santa Cruz, CA, USA; diluted to 1:100), TLR5 mouse monoclonal (IMG-664A, Imgenex, San Diego, CA, USA; diluted to 1:200), TLR7 rabbit polyclonal (IMG-581A, Imgenex, San Diego, CA, USA; diluted to 1:300), and TLR9 rabbit polyclonal (sc-25468, Santa Cruz Biotechnology, Santa Cruz, CA, USA; diluted to 1:100). Dako REAL enVision/HRP, Rabbit/Mouse (ENV) served as the secondary antibody, visualizing was performed with the Dako REAL DAB+ Chromogen and counterstaining with Meyer’s hematoxylin. Specimens processed without a primary antibody were used as the negative controls and tissue with a known high immunoreactivity to these antigens (tonsillar, skin, and cutaneous squamous cell carcinoma) served as positive controls.

### Immunohistochemistry for CD3 and CD8

The pretreatment and immunohistochemical staining for CD3 and CD8 were performed with automatic Roche Ventana BenchMark ULTRA equipment (F. Hoffman-La-Roche AG, Basel, Switzerland). The deparaffinization, rehydration, and antigen retrieval were performed by treating the slides for 64 min in a Ventana Cell Conditioning (CC1) solution. After pretreatment, the slides were incubated with primary antibodies: ready-to-use rabbit monoclonal CD3 antibody (Ventana, clone 2GV6) or mouse monoclonal CD8 antibody (Novocastra, clone 4B11; diluted to 1:50) for 40 min. Antibodies were then detected and visualized using the Ventana Ultraview DAB detection kit. Finally, the slides were counterstained with Meyer’s hematoxylin and washed in tap water. Tonsillar tissue, known to contain large amounts of CD3- and CD8-postitive cells, was used as the positive control. Specimens processed without primary antibodies were used as negative controls.

### Scoring of samples

The immunostainings of the tumor samples were scored independently by assessors (I.B.-L., J.H., and J.K.) blinded to the clinical data. In the case of differences in the scoring results between assessors, specific spots were re-evaluated and discussed until consensus was reached. In a few cases, the scoring failed due to missing tumor tissue or a technical failure.

The cytoplasmic immunopositivity of TLR2, TLR4, and TLR7 and the nuclear immunopositivity of TLR5 were scored on a four-point scale: the absence of staining was scored as 0, weak immunoactivity as 1, moderate as 2, and strong staining as 3. The highest score from four spots was used for further analysis. The TLR2 tissue immunoexpression was successfully interpreted in 541 cases (98.5%), TLR4 in 537 (97.8%), TLR5 in 539 (98.2%), and TLR7 in 539 (98.2%). For the final statistical analysis, the TLRs immunoexpression scores were dichotomized as follows: TLR2 and TLR4 as low (scores 0–1) and high (scores 2–3), TLR5 as low (0–2) and high (3), and TLR7 as negative (0) and positive (1–2) expression levels. The distribution of the scores and examples of immunostainings were reported elsewhere [19].

The CD3 and CD8 immunopositivity in the intratumoral area (CD3^T^ and CD8^T^) was scored on a four-point scale: the absence of positive cells was scored as 0, a few solitary individual positive cells as 1, small positive cell clusters (5% positive cells) as 2, and extensive and organized staining (more than 10% positive cells) as 3. A five-point scale was used for stromal CD3 (CD3^S^) and CD8 (CD8^S^): no positive cells was scored as 0, a few solitary positive cells as 1, individual scattered cells and small clusters (5% positive cells) as 2, medium clusters (10% positive cells) as 3, and extensive staining of cells (over 20% positive cells) as 4. CD3^T^ was successfully assessed in 516 cases (94.0%); and CD8^T^, CD3^S^, and CD8^S^ in 515 cases (93.8%). In some cases, the scoring failed due to a missing tumor tissue or technical failure. CD3^T^ and CD8^T^ densities were dichotomized as negative (score 0) and positive (scores 1–3); CD3^S^ and CD8^S^ were dichotomized to low (score 0–3) and high (score 4). The dichotomized CD3 and CD8 densities were used to determine the CD3–CD8 tumor–stroma index using principles resembling the renowned *immunoscore* [33]. One point was given for positive CD3^T^ and CD8^T^ densities and high CD3^S^ and CD8^S^ densities resulted in a four-point scale (points 0 to 4). The immunostaining examples and scoring distribution are summarized in a flowchart presenting the determination of CD3–CD8 tumor–stroma index (S1 Fig).

### Statistical analysis

The associations between different TLRs and CD3 and CD8 immune cells were calculated using the Pearson’s chi-square test, while correlations were determined using the Spearman’s correlation test. We created survival curves using the Kaplan–Meier method and compared different groups using the log-rank test. For survival rates, we calculated the 95% confidence intervals (CIs). The disease-specific survival (DSS) was calculated from the day of surgery until death due to CRC or until the end of follow-up. For all analyses, we considered a two-tailed *p* < 0.05 as statistically significant. All statistical analyses were performed using SPSS version 26.0 (IBM’s SPSS Statistics, version 26.0 for Mac; SPSS, Inc., Chicago, IL, USA).

## Results

### Associations and correlations between TLRs and CD3 and CD8 immune cell densities

An association emerged between a high TLR2 immunuoexpression and positive CD3^T^ (*p* < 0.001; chi-square test, Table 1) and CD8^T^ (*p* = 0.001; chi-square test; Table 1). Weak positive correlations were observed between the TLR2 immunoexpression and the CD3^T^ (r_s_ = 0.175; *p* < 0.001; Table 2) and CD8^T^ densities (r_s_ = 0.131; *p* = 0.003; Table 2).

**Table 1:** Associations between the immunoexpressions of TLRs and the tumoral and stromal densities of CD3 and CD8 cells and CD3–CD8 tumor–stroma index in 549 CRC patients.

	TLR2			TLR4			TLR5			TLR7		
	Low (%)	High (%)	*p* value^1^	Low (%)	High (%)	*p* value^1^	Mild (%)	High (%)	*p* value^1^	Negative (%)	Positive (%)	*p* value^1^
CD3^T^												
Negative	37 (20.7)	142 (79.3)	**<0.001**	76 (42.9)	101 (57.1)	**0.013**	122 (68.2)	57 (31.8)	**0.001**	26 (14.6)	152 (85.4)	0.116
Positive	27 (8.1)	306 (91.9)		106 (31.8)	227 (68.2)		176 (52.9)	157 (47.1)		33 (9.9)	299 (90.1)	
CD3^S^												
Low	51 (13.0)	341 (87.0)	0.547	142 (36.3)	249 (63.7)	0.631	244 (62.2)	148 (37.8)	**0.001**	49 (12.6)	341 (87.4)	0.215
High	13 (10.9)	106 (89.1)		40 (33.9)	78 (66.1)		54 (45.4)	65 (54.6)		10 (8.4)	109 (91.6)	
CD8^T^												
Negative	36 (17.9)	165 (82.1)	**0.001**	84 (42.0)	116 (58.0)	**0.025**	132 (65.3)	70 (34.7)	**0.011**	20 (9.9)	181 (90.1)	0.340
Positive	25 (8.1)	285 (91.9)		100 (32.3)	210 (67.7)		167 (54.0)	142 (46.0)		39 (12.7)	269 (87.3)	
CD8^S^												
Low	51 (12.6)	353 (87.4)	0.352	146 (36.2)	257 (63.8)	0.840	241 (59.7)	163 (40.3)	0.309	42 (10.4)	361 (89.6)	0.116
High	10 (9.3)	97 (90.7)		38 (35.5)	69 (64.5)		58 (54.2)	49 (45.8)		17 (15.9)	90 (84.1)	
CD3–CD8 tumor–stroma index												
0	26 (24.3)	81 (75.7)	**<0.001**	52 (49.1)	54 (50.9)	**0.022**	77 (72.0)	30 (28.0)	**0.003**	17 (15.9)	90 (84.1)	0.057
1	8 (7.4)	100 (92.6)		33 (30.3)	76 (69.7)		68 (62.4)	41 (37.6)		11 (10.1)	98 (89.9)	
2	14 (9.5)	134 (90.5)		48 (32.7)	99 (67.3)		80 (54.4)	67 (45.6)		15 (10.3)	131 (89.7)	
3	6 (6.7)	83 (93.3)		27 (30.3)	62 (69.7)		41 (46.1)	48 (53.9)		6 (6.7)	83 (93.3)	
4	4 (8.9)	41 (91.1)		17 (37.8)	28 (62.2)		23 (51.1)	22 (48.9)		10 (22.2)	35 (77.8)	

Abbreviations: CD3, CD3-positive immune cell; CD8, CD8-positive immune cell; TLR, toll-like receptor.

^1^Chi-square test.

**Table 2:** Correlations between the immunoexpressions of TLRs and the tumoral and stromal densities of CD3 and CD8 cells and CD3–CD8 tumor–stroma index in 549 CRC patients.

	TLR2		TLR4		TLR5		TLR7	
	r_s_	*p* value	r_s_	*p* value	r_s_	*p* value	r_s_	*p* value
CD3^T^	0.175	**<0.001**	0.135	**0.002**	0.206	**<0.001**	0.095	**0.031**
CD3^S^	0.050	0.262	0.014	0.759	0.137	**0.002**	0.065	0.142
CD8^T^	0.131	**0.003**	0.117	**0.008**	0.140	**0.002**	-0.008	0.852
CD8^S^	0.072	0.104	0.012	0.791	0.052	0.235	-0.057	0.197
CD3–CD8 index	0.157	**<0.001**	0.098	**0.029**	0.203	**<0.001**	0.032	0.477

Abbreviations: CD3, CD3-positive immune cell; CD8, CD8-positive immune cell; TLR, toll-like receptor.

r_s_ = Spearmans’s correlation coefficent.

A high TLR4 immunoexpresssion associated with positive CD3^T^ (*p* = 0.013; chi-square test; Table 1) and CD8^T^ (*p* = 0.025; chi-square test; Table 1). Weak positive correlations emerged between the TLR4 immunoexpression and CD3^T^ (r_s_ = 0.135; *p* = 0.002; Table 2) and CD8^T^ (r_s_ = 0.117; *p* = 0.008; Table 2).

In addition, we observed an association between a low TLR5 immunoexpression and negative CD3^T^ (*p* = 0.001; chi-square test; Table 1), low CD3^S^ (*p* = 0.001; chi-square test; Table 1), and negative CD8^T^ (*p* = 0.011; chi-square test; Table 1). Weak positive correlations emerged between the TLR5 immunoexpression and CD3^T^ (r_s_ = 0.206; *p* < 0.001; Table 2), CD3^S^ (r_s_ = 0.137; *p* = 0.002; Table 2), and CD8^T^ (r_s_ = 0.140; *p* = 0.002; Table 2).

We found no association or correlation between TLR7 immunoexpressions and CD3 or CD8 cell densities.

### Associations and correlations between TLRs and the CD3–CD8 tumor–stroma index

A high tissue TLR2 immunoexpression associated with a higher CD3–CD8 tumor–stroma index (*p* < 0.001; chi-square test; Table 1), with a weak positive correlation (r_s_ = 0.157; *p* < 0.001; Table 2).

A high TLR4 immunoexpression associated with a higher CD3–CD8 tumor–stroma index (*p* = 0.022, chi-square test; Table 1), with a positive correlation (r_s_ = 0.098; *p* = 0.029; Table 2).

In addition, an association emerged between a low TLR5 immunoexpression and a lower CD3–CD8 tumor–stroma index (*p* = 0.003; chi-square test; Table 1) alongside a weak positive correlation (r_s_ = 0.203; *p* < 0.001; Table 2).

We observed no association or correlation between the TLR7 immunoexpression and the CD3–CD8 tumor–stroma index.

### Survival analysis

**Fig 1:**
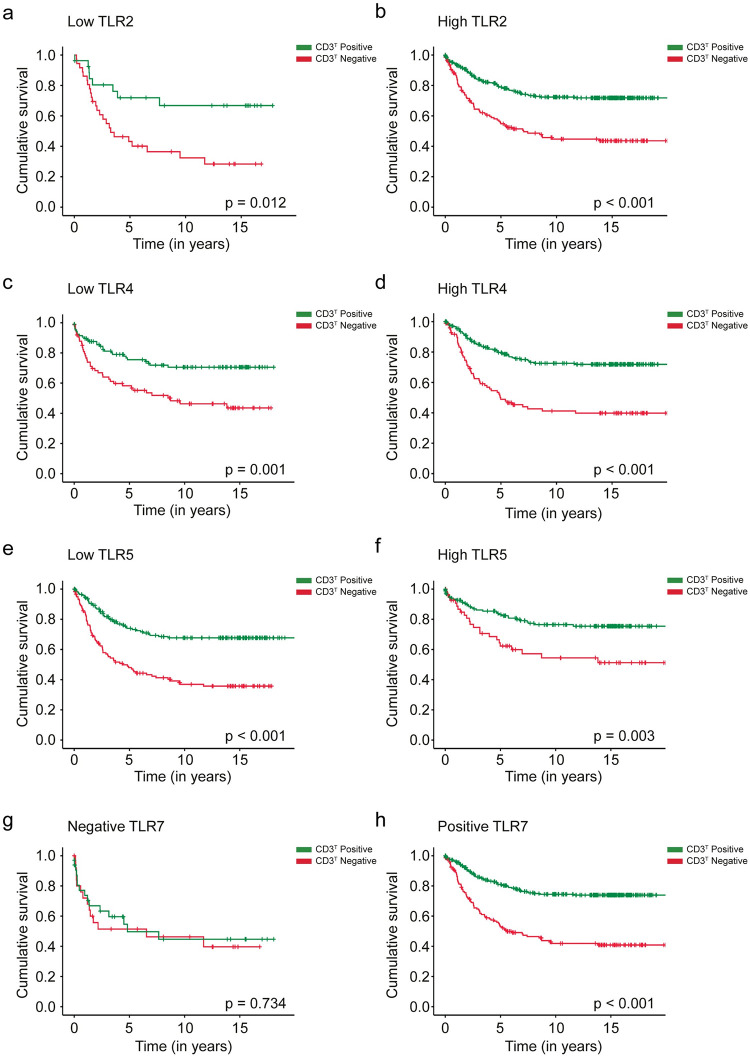
Disease-specific survival analysis of colorectal cancer patients using the Kaplan–Meier method. A negative versus positive intratumoral CD3^T^ expression among (**a**) low TLR2 expression patients, (**b**) high TLR2 expression patients, (**c**) low TLR4 expression patients, (**d**) high TLR4 expression patients, (**e**) low TLR5 expression patients, (**f**) high TLR5 expression patients, (**g**) TLR7-negative patients, and (**h**) TLR7-positive patients. The log-rank test was used.

Patients with a positive CD3^T^ exhibited a significantly better prognosis among subgroups with a low TLR2 [hazard ratio (HR) 0.36; 95% CI 0.16–0.79; *p* = 0.012; Fig 1a] and a high TLR2 immunoexpression (HR 0.40; 95% CI 0.29–0.55; *p* < 0.001; Fig 1b). Patients with a positive CD3^T^ experienced a significantly better prognosis among subgroups with a low TLR4 (HR 0.45; 95% CI 0.27–0.72; *p* = 0.001; Fig 1c) and a high TLR4 expression (HR 0.35; 95% CI 0.24–0.51; *p* < 0.001; Fig 1d). Similarly, patients with a positive CD3^T^ exhibited a better prognosis among subgroups with a low TLR5 (HR 0.38; 95% CI 0.26–0.54; *p* < 0.001; Fig 1e) and a high TLR5 expression (HR 0.45; 95% CI 0.26–0.76; *p* = 0.003; Fig 1f). Among positive CD3^T^ patients, those with a positive TLR7 immunoexpression exhibited a significantly better prognosis (HR 0.34; 95% CI 0.24–0.47; *p* < 0.001; Fig 1h).

**Fig 2:**
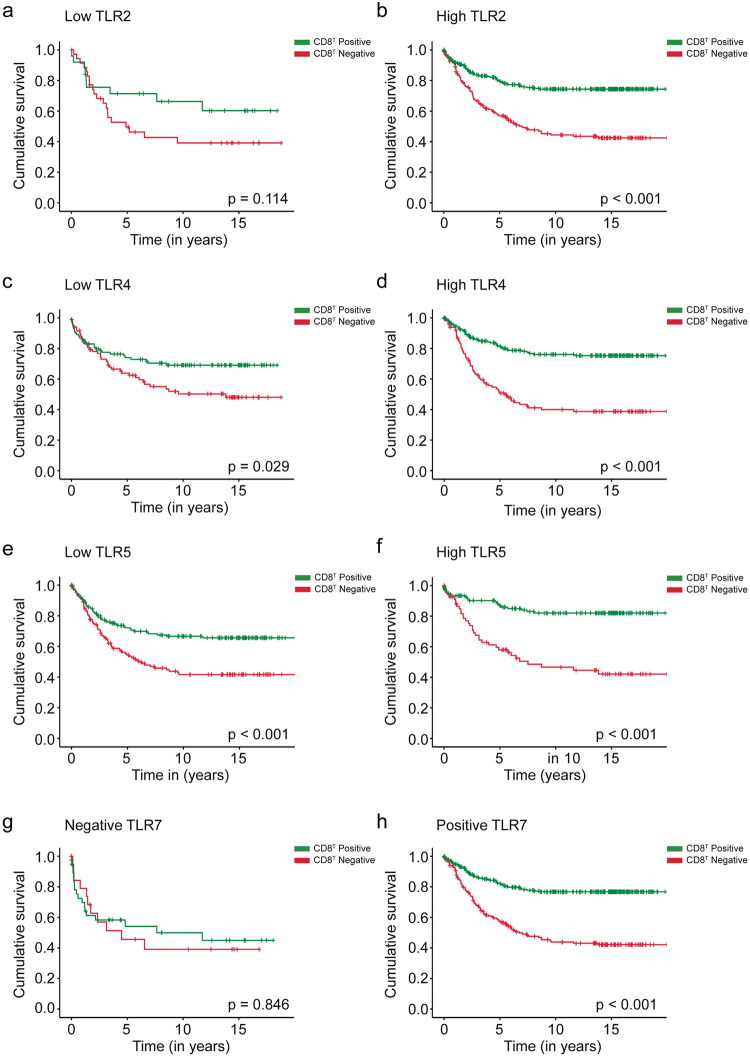
Disease-specific survival analysis of colorectal cancer patients using the Kaplan–Meier method. A negative versus positive intratumoral CD8^T^ expression among (**a**) low TLR2 expression patients, (**b**) high TLR2 expression patients, (**c**) low TLR4 expression patients, (**d**) high TLR4 expression patients, (**e**) low TLR5 expression patients, (**f**) high TLR5 expression patients, (**g**) TLR7-negative patients, and (**h**) TLR7-positive patients. The log-rank test was used.

Patients with a positive CD8^T^ exhibited a better prognosis among subgroups with a high TLR2 expression (HR 0.38; 95% CI 0.27–0.52; *p* < 0.001; Fig 2b), a low TLR4 expression (HR 0.59; 95% CI 0.36–0.95; *p* = 0.029; Fig 2c), a high TLR4 expression (HR 0.31; 95% CI 0.21–0.45; *p* < 0.001; Fig 2d), a low TLR5 expression (HR 0.51; 95% CI 0.36–0.74; *p* < 0.001; Fig 2e), a high TLR5 expression (HR 0.25; 95% CI 0.15–0.43; *p* < 0.001; Fig 2f), and a positive TLR7 expression (HR 0.32; 95% CI 0.23–0.45; *p* < 0.001; Fig 2h). Survival analysis of low versus high stromal CD3 and CD8 expression among TLR subgroups showed similar results as intratumoral CD3 and CD8 expressions (S2 and S3 Figs).

**Fig 3:**
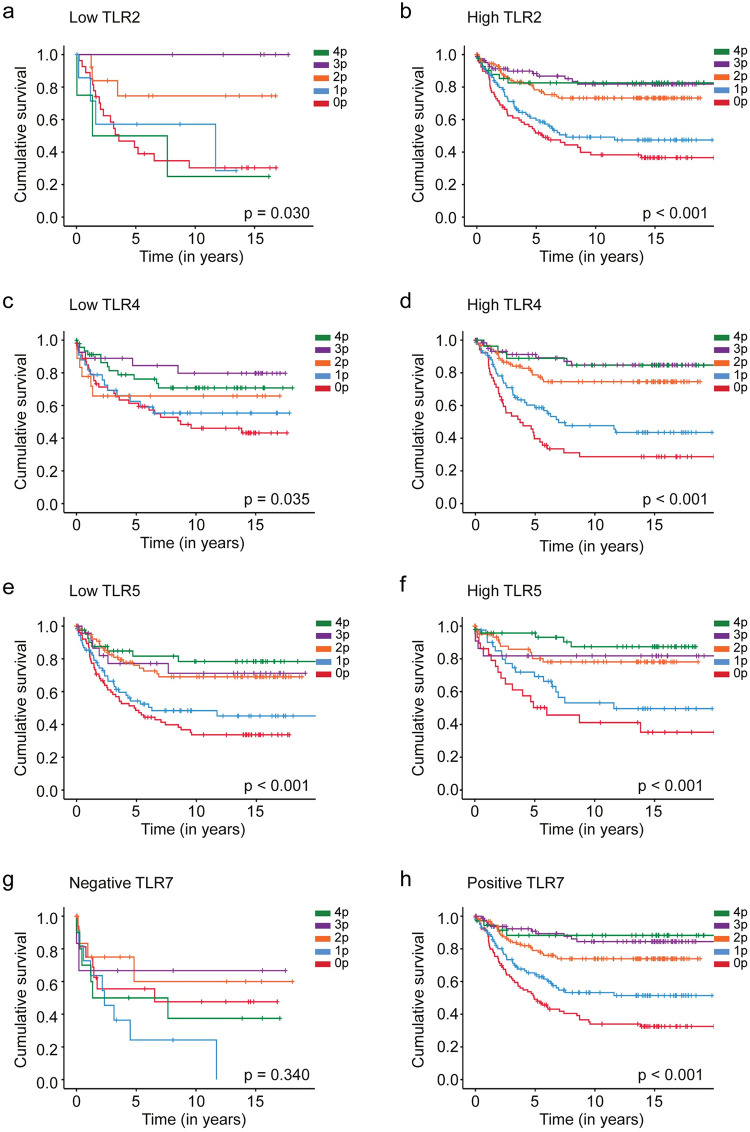
Disease-specific survival analysis of colorectal cancer patients using the Kaplan–Meier method. CD3–CD8 five-point index among (**a**) low TLR2 expression patients, (**b**) high TLR2 expression patients, (**c**) low TLR4 expression patients, (**d**) high TLR4 expression patients, (**e**) low TLR5 expression patients, (**f**) high TLR5 expression patients, (**g**) TLR7-negative patients, and (**h**) TLR7-positive patients. The log-rank test was used.

Among all TLR2, TLR4, and TLR5 subgroups and the positive TLR7 subgroup, patients with a low CD3–CD8 tumor–stroma index exhibited a worse survival (Fig 3). Among those with a high TLR2 expression, patients with a CD3–CD8 tumor–stroma index of 4 s had a five-year DSS of 82.2% (95% CI 70.2–94.2) compared to 51.8% (95% CI 40.6–63.0; *p* < 0.001, log-rank test; Fig 3b) among patients with an index of 0. In the high TLR4 expression subgroup, five-year DSS reached 88.9% (95% CI 77.1–100.0) among patients with the highest CD3–CD8 tumor–stroma index, falling to 39.7% (95% CI 26.4–53.0; *p* < 0.001, log-rank test; Fig 3d) among patients with the lowest CD3–CD8 tumor–stroma index. Among patients with a high TLR5 expression, five-year DSS was 81.8% (95% CI 65.7–97.9) among those with a CD3–CD8 tumor–stroma index of 4, falling to 50.3% (95% CI 31.9–68.7; *p* < 0.001, log-rank test; Fig 3f) among patients with the lowest CD3–CD8 tumor–stroma index. Among TLR7-positive patients, those with the highest CD3–CD8 tumor–stroma index had a five-year DSS of 87.9% (95% CI 76.7–99.1) compared to 48.0% (95% CI 37.4–58.6; *p* < 0.001, log-rank test; Fig 3h) among patients with the lowest CD3–CD8 tumor–stroma index.

## Discussion

To our knowledge, the relationship between tissue immunoexpression of TLRs and CD3- or CD8-positive immune cells has not been previously studied among CRC patients. Here, we found that among patients with high immunoexpressions of the TLRs investigated, patients with a high CD3–CD8 tumor–stroma index exhibited a better prognosis. Positive intratumoral CD3^T^ and CD8^T^ levels, and a high CD3–CD8 tumor–stroma index associated and correlated with a high TLR2 and a high TLR4 immunoexpression. A low TLR5 immunoexpression associated and correlated with negative intratumoral CD3^T^ and CD8^T^ levels, low stromal CD3^S^ and CD8^S^ levels, and a low CD3–CD8 tumor–stroma index. We observed no association between the TLR7 immunoexpression and CD3 or CD8 densities or a CD3–CD8 tumor–stroma index, and only a weak correlation with a positive CD3^T^.

CD3- and CD8-positive cells are crucial in the adaptive antitumoral immune response, modulating cancer outcomes [22]. In our previous study, high CD3^T^, CD3^S^, CD8^T^, and CD8^S^ levels emerged as markers of a good prognosis in CRC patients [27]. The *immunoscore*, a combination of CD3 and CD8 immune cell densities in the central and peripheral tumor, enhances the prognostic value [24, 34, 35]. We found that CD3^T^, CD3^S^, CD8^T^, and CD8^S^ positivity indicated a better prognosis or carried no prognostic value in different TLR subgroups, suggesting that the expression of some TLRs might influence the prognostic value of these TILs. The additional prognostic value of CD8 densities remains debatable. We did not find any TLR subgroups, where CD8^T^ or CD8^S^ carries a prognostic significance, but CD3^T^ or CD3^S^ does not. Furthermore, the CD3–CD8 tumor–stroma index did not have a significant prognostic value in any additional TLR subgroups compared to CD3^T^. Interestingly, CD3^T^ positivity indicated a better prognosis even among low TLR2 patients, whereas CD8^T^ carried no prognostic significance in this subgroup. Similarly, among patients with a high TLR5 expression, a high CD3^S^ indicated a better prognosis, while CD8^S^ did not serve as a significant prognostic factor in this subgroup. This could suggest that the TLR expression—namely, TLR2 and TLR5—might modulate the antitumorigenic effect of CD8-positive cells but would not carry such an effect on the prognostic role of CD3-positive cells.

Previously, the connection between innate and adaptive inflammation in malignant tissue has been investigated in well-differentiated follicular thyroid carcinoma. Both a strong TLR4 expression and the lack of TLR4 expression emerged as markers of aggressive disease, and those patients with metastasized disease had fewer CD45+ lymphocytes around the tumor compared to patients without metastases [36]. Interestingly, among patients with a high CRP, a high TLR4 immunoexpression associated with a better prognosis in our previous study [19]. In this study, the CD3–CD8 tumor–stroma index had a quite pronounced effect on survival among high TLR4 patients. Almost 90% of patients with the highest CD3–CD8 tumor–stroma index were alive after five years, but among those with the lowest index less than 40% survived the same time period.

In another study, the proinflammatory markers CD68, CD15, IL-6, and TLR4 were upregulated in colorectal adenocarcinoma compared to normal mucosa or premalignant conditions [37]. In that study, patients with a high TLR4 expression experienced an earlier relapse than those with a low TLR4 expression. Similarly, in our previous study, a high TLR4 associated with a worse prognosis in Dukes B CRC patients [17]. In addition, CD68 is expressed on antigen-presenting cells, cells connecting innate and adaptive immunity, possibly explaining the upregulation of CD68 in their work [38].

Väyrynen et al. investigated components of the innate and adaptive immune systems and antigen-presenting cells in CRC tissue samples. Mature CD83 dendritic cells clustered with CD3 T-cells and associated with a lower stage of disease. The association between a higher Klintrup–Mäkinen score and higher densities of CD3 and CD8 cells in intratumoral and peritumoral areas were also observed [39]. Similarly to our findings here, the associations between the innate and adaptive immune systems emerged, although we investigated TLRs as innate immune response components.

TLRs expressed on antigen-presenting cells, but also on different T-cells, act as costimulatory components in T-cell activation [12, 15]. Interestingly, various TLRs are represented in different cell subtypes [12]. The variation and amount of expressed TLRs depend on several factors, while various TLRs play different roles in T-cell activation. For example, TLR5 and TLR4 enhance the suppressive function of Treg cells, while TLR2 and TLR7 lead to Treg cell proliferation and the blocking of the suppressive role [12, 15]. Natural killer T-cells (NKTs), however, bridge innate and adaptive immune responses. Following TLR stimulation, antigen-presenting cells stimulate invariant NKTs (iNKTs) by producing several cytokines. However, iNKTs can also be activated directly by TLRs [40, 41]. TLR expression by iNKTs can be induced by CD3 stimulation and IFN-a [40], demonstrating that TLRs and T-cells have continuous two-sided communication. Specifically, TLRs induce T-cell differentiation and activation, in line with the positive correlation between most TLRs and TILs our study revealed, and, conversely, activated T-cells impact the TLR expression.

The innate and adaptive immune systems work together to detect and fight against a developing tumor [42]. TLRs are needed for DC maturation, T-cell maturation and activation, as well as for adaptive immune responses. Certain TLRs might allure the T-cells to the tumour area. Cytokines produced by T-cells may promote tumor cells or other components of the tumor microenvironment to develop adaptive immune resistance by expressing proteins and producing cytokines, thereby escaping the host’s antitumor immune mechanisms [43]. The TLR protumorigenic role likely influences and/or downregulates the adaptive immune system components [44]. Dysfunctional TLR signaling leads to the invasion of tumor cells by inducing the epithelial-to-mesenchymal transition, which in a normal environment is a mechanism used by lymphocytes to move to inflammatory areas. But, in malignancies, tumor cells learn to use the host’s normal biological mechanisms to their benefit [44]. In our current work, positive correlations between several TLRs and TILs were observed, supporting the idea that imbalanced TLR signaling leads to imbalanced adaptive immune responses.

One strength of our study is its large, well-characterized cohort with reliable survival data and a long follow-up time. The single-center setting may represent a limitation, whereby further multicenter studies are needed to validate our results. Using TMA slides can be seen as a limitation since a smaller proportion of the tumor is evaluated compared to whole slides. However, previous studies have demonstrated that the TMA technique is sufficiently representative of the tumor [45]. Yet, the TMA technique provides us with an opportunity to investigate more samples faster and to retain valuable tumor tissue for further studies.

To our knowledge, this is the first study to investigate the prognostic value of CD3- and CD8-positive immune cells in different TLR subgroups in CRC. We demonstrate here that the tissue expression of several TLRs associated and correlated with CD3^T^, CD3^S^, CD8^T^, and CD8^S^ positivity. Further research is needed to identify the biological mechanisms behind the relationships between innate and adaptive immune responses.

## Supporting information

S1 Fig Flowchart of the determination of the CD3–CD8 tumor–stroma index and representative images of dichotomized CD3 and CD8 immunostainings. Original magnification: x20.
(TIF)

S2 Fig Disease-specific survival analysis of colorectal cancer patients using the Kaplan–Meier method. A low versus high stromal CD3^S^ expression among (**a**) low TLR2 expression patients, (**b**) high TLR2 expression patients, (**c**) low TLR4 expression patients, (**d**) high TLR4 expression patients, (**e**) low TLR5 expression patients, (**f**) high TLR5 expression patients, (**g**) TLR7-negative patients, and (**h**) TLR7-positive patients. The log-rank test was used.
(TIF)

S3 Fig Disease-specific survival analysis of colorectal cancer patients using the Kaplan–Meier method. A low versus high stromal CD8^S^ expression among (**a**) low TLR2 expression patients, (**b**) high TLR2 expression patients, (**c**) low TLR4 expression patients, (**d**) high TLR4 expression patients, (**e**) low TLR5 expression patients, (**f**) high TLR5 expression patients, (**g**) TLR7-negative patients, and (**h**) TLR7-positive patients. The log-rank test was used.
(TIF)

S1 Table Clinicopathological characteristics of 549 CRC patients. (DOCX)

## Abbreviations

CI: confidence interval
CRC: colorectal cancer
CRP: C-reactive protein
DAMP: damage-associated molecular pattern
DSS: disease-specific survival
HR: hazard ratio
IQR: interquartile range
mGPS: modified Glasgow prognostic score
NK: natural killer cell
NLR: neutrophil–lymphocyte ratio
PAMP: pathogen-associated molecular pattern
SIR: systemic inflammatory response
TIL: tumor-infiltrating lymphocyte
TLR: toll-like receptor
TMA: tissue microarray
