# Supplementary figures and images for: High tissue expression of TLRs combined with high density of tumor infiltrating lymphocytes predicts a better prognosis in colorectal cancer patients
Source: PLoS One. 2023 Jan 17;18(1):e0280085. doi: 10.1371/journal.pone.0280085 (PMC9844887; doi:10.1371/journal.pone.0280085)

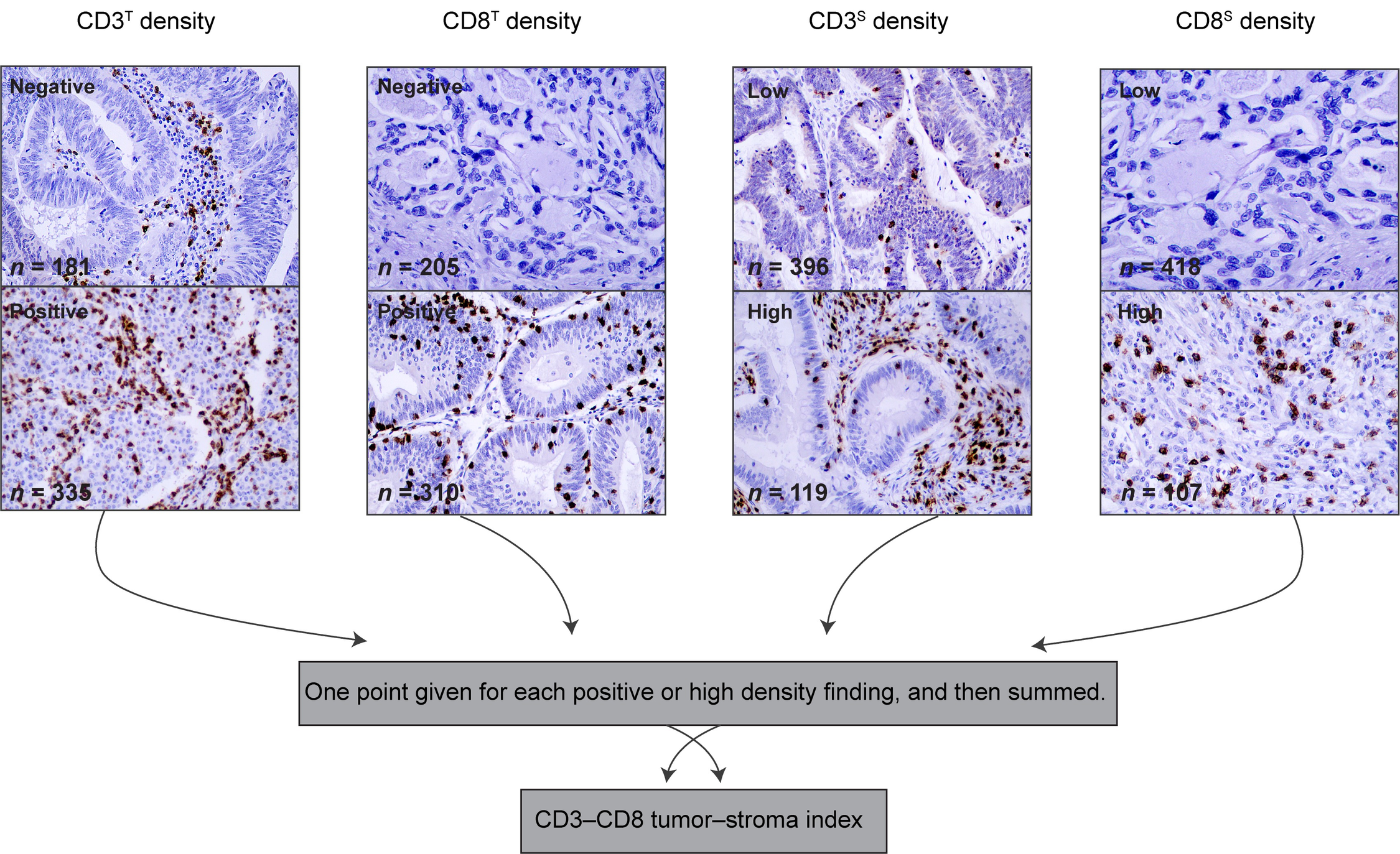

Supplement: S1 Fig — Original magnification: x20. (TIF) [file pone.0280085.s001.tif]

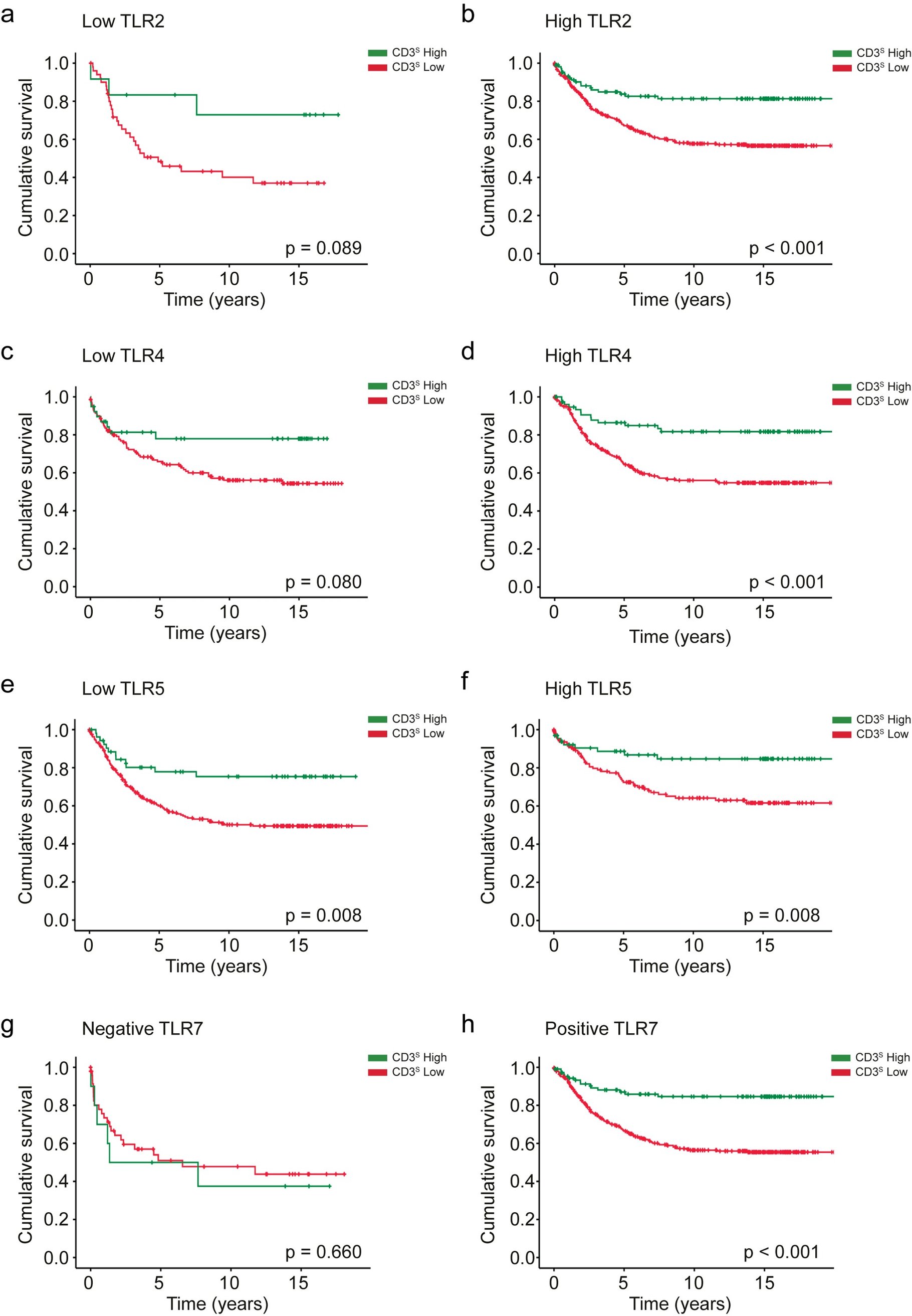

Supplement: S2 Fig — A low versus high stromal CD3S expression among (a) low TLR2 expression patients, (b) high TLR2 expression patients, (c) low TLR4 expression patients, (d) high TLR4 expression patients, (e) low TLR5 expression patients, (f) high TLR5 expression patients, (g) TLR7-negative patients, and (h) TLR7-positive patients. The log-rank test was used. (TIF) [file pone.0280085.s002.tif]

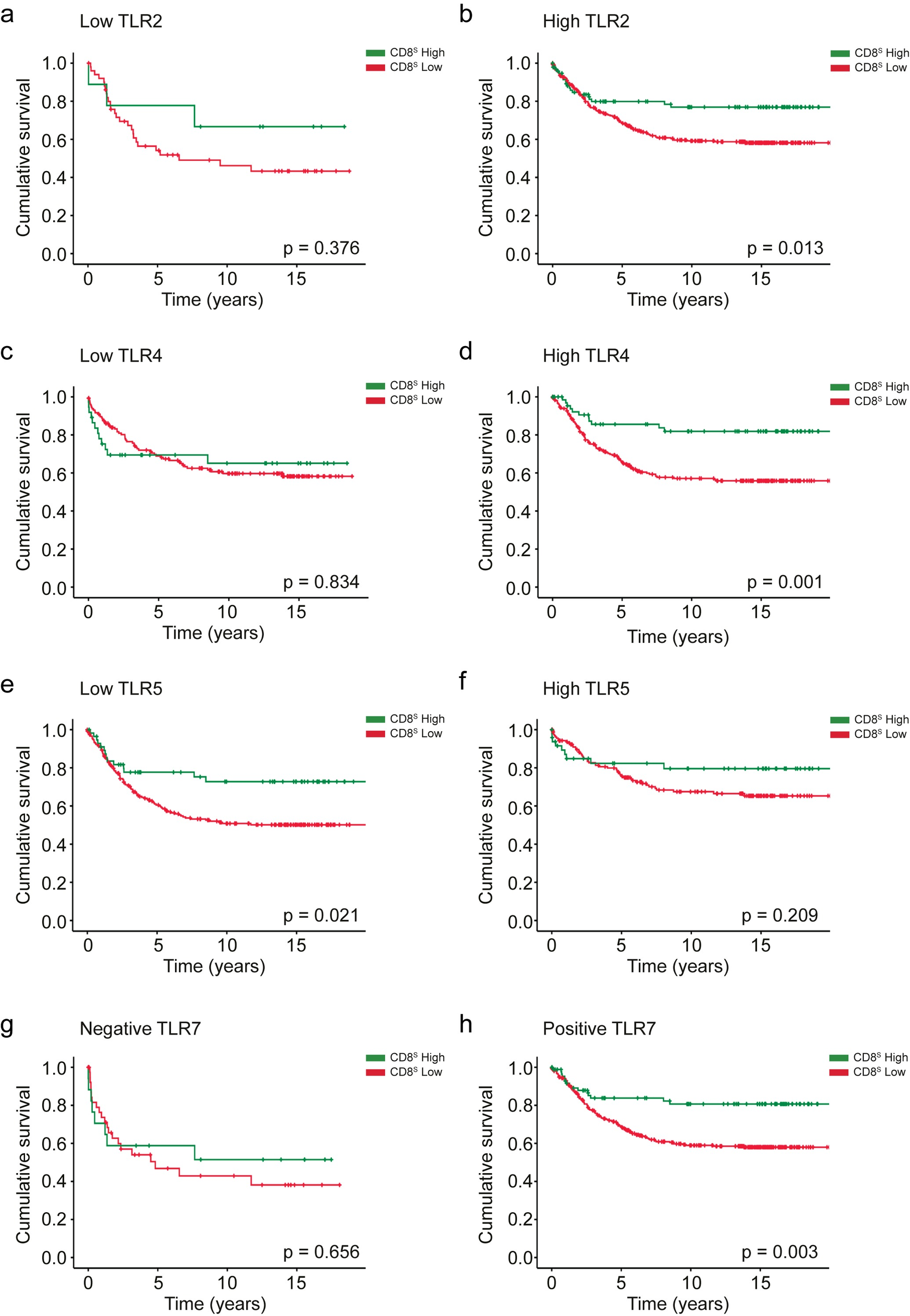

Supplement: S3 Fig — A low versus high stromal CD8S expression among (a) low TLR2 expression patients, (b) high TLR2 expression patients, (c) low TLR4 expression patients, (d) high TLR4 expression patients, (e) low TLR5 expression patients, (f) high TLR5 expression patients, (g) TLR7-negative patients, and (h) TLR7-positive patients. The log-rank test was used. (TIF) [file pone.0280085.s003.tif]
