# Supplementary material for: High tissue expression of TLRs combined with high density of tumor infiltrating lymphocytes predicts a better prognosis in colorectal cancer patients
Source: PLoS One. 2023 Jan 17;18(1):e0280085. doi: 10.1371/journal.pone.0280085 (PMC9844887; doi:10.1371/journal.pone.0280085)
Supplement: S1 Table — (DOCX) [file pone.0280085.s004.docx]

Table S1. Clinicopathological characteristics

of 549 CRC patients

| **Clincopathological** | | |
| --- | --- | --- |
| **variable** |  | **n (%)** |
| Age |  |  |
| <65 |  | 220 (40.1) |
| ≥65 |  | 329 (59.9) |
| Gender |  |  |
| Male |  | 289 (52.6) |
| Female |  | 260 (47.4) |
| Location |  |  |
| Colon | | 281 (51,2) |
| Rectum |  | 268 (48.8) |
| Tumor stage |  |  |
| I |  | 108 (19.7) |
| II |  | 153 (27.9) |
| III |  | 201 (36.7) |
| IV |  | 86 (15.7) |
| Tumor classification (pT) | | |
| pT1–pT2 |  | 134 (24.8) |
| pT3–pT4 |  | 407 (75.2) |
| Lymph node metastases (pN) | | |
| pN0 |  | 276 (51.2) |
| pN1–2 |  | 263 (48.8) |
| Tumor grade (WHO) | | |
| 1–2 |  | 432 (87.6) |
| 3–4 |  | 61 (12.4) |
